# The Increasing Frequency of the Use of Narcotic Drugs by Members of the Medical Profeesion and the Probable Reasons for It

**Published:** 1910-04

**Authors:** W. C. Ashworth

**Affiliations:** Greenesboro, N. C.; Resident Physician, Telfair Sanitarium


### THE INCREASING FREQUENCY OF THE USE OF NARCOTIC DRUGS BY MEMBERS OF THE MEDICAL PROFESSION AND THE PROBABLE REASONS FOR IT.

By W. C. Ashworth, M. D., Greenesboro, N. C.

Resident Physician, Telfair Sanitarium.

The average physician is not aware of the appalling extent to which the members of our profession are addicted to the use of narcotic drugs. My line of work gives me ample opportunity to note the increasing evil to a greater degree than is imagined or even thought of by the general practitioner or layman. We find on loking over our case register that 75 per cent. of all our drug patients are physicians. This per cent. is perhaps a little larger than is found in the average institution, which may be accounted for in part, by our direct method of advertising to the profession; however, we do not believe that it is much larger than is found in most institutions.

I wish to note briefly the causes which are responsible for this evil which has befallen so many of our medical brethren. It is an evil which is enslaving a large and increasing percentage of the best men in our profession.

The first and leading cause we ascribe to the meager teaching in our medical colleges of the baneful effects of the habitual use of narcotic drugs. The average mdical student leaves college with but a slight idea of the insidious action of morphine and cocaine or the easy avenue which leads to the contraction of the habit. If the students of our medical colleges were well grounded in this important branch of medicine, it would not only perhaps be their own salvation, but would in a measure stop the indiscriminate prescribing of narcotic drugs to their patients. Among the questions to the physician entering our institution for treatment he is asked how he contracted the morphine or cocaine habit and he almost invariably answers that he commenced its use for acute pain or for the relief of fatigue caused by overwork, not realizing any more than the average layman the insidious action of the drug. I regard it as a great stigma on our profession that such a condition of affairs exists, but on the other hand, I hold that the unfortunate habitue is less blameable than his teachers, whose duty it was to have given him more information on a subject that so widely concerned his future success in the most trying of all professions.

Now as to the remedy, again I would suggest that a special course of lectures be given each class of medical students on the narcotic drugs and their habit forming tendencies in order that the student may leave college and take up his work with a clear and concise knowledge of the action of these drugs and know that if they are habitually used they are a constant menace to the life and mentality of the user and that sure and certain dire results must inevitably follow in their wake.

The laity have very little patience with the physician who is addicted to the use of drugs. They say at once, that he above all others is inexcusable, because he knew the action of the drug before commencing its use. I am sorry also to note that the members of the medical profession have about as little patience with the habituated physician as the average layman has. Now the remedy lies in the proper teaching of this subject in our medical colleges and secondly with the individual himself. The physician must not entertain the idea that he is immune to the temptations of other people or that his will-power is of a superior nature to that of his layman brother, per contra we generally find just the reverse of this condition of affairs. On account of his irregular hours and the constant grind on his nervous system, incident to his laborious profession, he is really more susceptible to the action of narcotic drugs and the relief from worry and overwork is more quickly sought, before he even realizes the danger and subtleness that lurks in the use of these drugs.

In defense of the unfortunate drug user, I wish to say that very few of them comemnce the use of the drug wantonly. Somewhere, perhaps in the ancestry of the individual, you will find the history of excessive use of stimulants or narcotic drugs, thus transmitting to the unfortunate subject a lessened resistance or a strong predilection for the use of stimulants and drugs. I find in support of this argument that fully 50 per cent. of my drug patients give a history of a depraved ancestry or other faulty habits of living, due in a large part to the excesisve use of narcotic drugs or stimulants. It is natural, therefore, to find the neuropathic type or tendency in most drug users.

I wish in this connection to reiterate what I stated about physicians taking narcotic drugs without any more knowledge of their habit-forming tendencies than the average layman and to this is added the constant danger of these drugs on account of their being handled daily by the physician, which places him in infinitely more danger than the average layman. When we realize fully the force of this truth we become more sympathetic and have more patience with the unfortunate physician who becomes addicted to the use of narcotic drugs.

Most people who handle explosives have received instructions as to their danger and certain precautions are given for their use, but not so with the physicians who daily handle narcotic drugs, which are even more dangerous than the most deadly explosive. The physician leaves his alma mater with the fixed belief that he can handle and perhaps take a medicinal dose of any drug with impunity and that he is or can always be master of the situation. This belief, perhaps, continues until he has taken a few doses of morphine during some acute illness for the relief of pain, for which time and rest would have been sufficient, but unfortunately he could spare neither, and when the illness has subsided he suddenly wakes up to the fact that it is impossible for him to live without the powerful anodyne and realizes as never before that there is no such salvery as the thralldom of a drug. Try as he may, to break the fetters that bind him he finds himself utterly helpless and wholly unable to cope with the situation. There is an old adage that a habit soon becomes second nature, but when it comes to the use of narcotic drugs it stands first and nature is in abeyance and all the forces of the body are insufficient to throw it off.

I wish to emphasize again, in view of the deplorable condition of affairs, that the failure of our medical teachers to teach every graduating class in medicine the insidiousness of narcotic drugs whether they be taken by the doctor himself or givn to his patient is well nigh criminal. When the physician learns this lesson well as he should, he will cease to give so much to his patients and conseqeuntly we shall have fewer morphine habitues among the laity. As an illustration of this point, I frequently elicit a history like this from a patient who is just entering for treatment. When questioned as to how he contracted the habit, he states he was sick and the doctor visited him once a day perhaps and administered a hypodermic for a period of days and possibly weeks, and when the illness subsided, the physician in attendance stated it would not be necessary to give any more hypodermics and by all means not to take any more himself. What happens? The patient writhes in agony when his accustomed narcotic is withdrawn and he forthwith sends for his regular medical adviser and is informed by him that the pain is largely imaginary and that there is really no necessity for his continuing the anodyne hypodermic. The poor patient implores and beseeches his doctor just for one more, but is stoutly refused. What is the result? The patient leaves his bed, if he is able, but if not, sends to the nearest drug store and arms himsellf with a hypodermic for future use. The future of this patient need not be pictured as he now belongs to the great rank and file of morphine users. Who says the poor fellow is to be blamed? Who is to be blamed most, the patient or his ill informed medical adviser? I tell you, gentlemen, we deal entirely too lightly with this question. Something must be done and that quickly, or we shall become a nation and a profession of drug users.

To my mind, the solution of the problem is to educate the physician himself, who will in turn save himself, as well as his patient, from the clutches of the "king" of all drugs, morphine. If I have succeeded in arousing your interest concerning this very important subject I shall feel more than repaid for the time spent in writing this hastily prepared article.

